# Circulating inflammation-related proteome improves cardiovascular risk prediction. Results from two large European cohort studies

**DOI:** 10.1007/s10654-025-01285-y

**Published:** 2025-08-13

**Authors:** Ruijie Xie, Sha Sha, Hermann Brenner, Ben Schöttker

**Affiliations:** 1 Division of Clinical Epidemiology and Aging Research, German Cancer Research Center, Im Neuenheimer Feld 581, 69120 Heidelberg, Germany; 2 Faculty of Medicine, Heidelberg University, 69115 Heidelberg, Germany

**Keywords:** Cardiovascular disease, Inflammation, Proteomics, Risk prediction, Proteins

## Abstract

**Background:**

Inflammation plays a crucial role in cardiovascular disease (CVD), but the value of inflammation-related proteins in predicting major adverse cardiovascular events (MACE) is unclear. This study evaluated whether incorporating inflammation-related proteins into the SCORE2 model improves 10-year MACE risk prediction.

**Methods:**

This study included 47,382 participants from the UK Biobank and 4,135 participants from the German ESTHER study without prior CVD or diabetes. We tested C-reactive protein (CRP) and 73 inflammation-related proteins measured with Olink^®^ panels. Biomarker selection was performed using least absolute shrinkage and selection operator (LASSO) regression with bootstrapping separately for males and females. Selected proteins were added to the SCORE2 model variables. Model performance was evaluated using Harrell’s C-index, net reclassification index (NRI), and integrated discrimination index (IDI).

**Results:**

Seven inflammation-related proteins but not CRP were selected, including two for both sexes, three specifically for males, and two specifically for females. Incorporating these proteins significantly improved the C-index (95% confidence interval (95%CI)) of the refitted SCORE2 model from 0.716 (0.698, 0.734) to 0.750 (0.732, 0.768) in internal validation in the UK Biobank and from 0.677 (0.644, 0.710) to 0.713 (0.681, 0.745) in external validation in the ESTHER study. The NRI with 95%CI was 12.4% (5.2%, 16.3%) in internal validation and 4.2% (0.5%, 23.6%) in external validation. The IDI also improved significantly.

**Conclusion:**

Incorporating inflammation-related proteins into the SCORE2 model significantly improves the prediction of 10-year MACE risk among individuals without prior CVD or diabetes. Measuring these proteins may enhance risk stratification in clinical practice.

**Supplementary Information:**

The online version contains supplementary material available at 10.1007/s10654-025-01285-y.

## Introduction

Cardiovascular disease (CVD) remains a leading cause of morbidity and mortality globally, with its burden expected to escalate due to aging populations and the increasing prevalence of risk factors such as obesity, hypertension, and unhealthy lifestyles [1, 2]. Accurately identifying individuals at high risk for major adverse cardiovascular events (MACE) is crucial for implementing timely and effective prevention measures [3].

In 2021, the SCORE2 model was introduced as a sex-specific tool for estimating 10-year MACE risk in adults without prior CVD or diabetes [4]. Derived from extensive data encompassing multiple European cohorts, SCORE2 incorporates traditional risk factors such as age, sex, blood pressure, cholesterol levels, and smoking status. However, no inflammation measure was included. While SCORE2 represents a significant advancement over earlier models, its predictive accuracy remains suboptimal, highlighting the need for additional biomarkers to refine risk estimation and improve early intervention strategies [5, 6].

Inflammation plays a pivotal role in the pathogenesis of CVD, contributing to atherosclerotic plaque formation, progression, and rupture [7]. Circulating inflammation-related proteins serve as biomarkers of vascular inflammation and have been associated with incident cardiovascular events [8–10]. C-reactive protein (CRP) is one such biomarker that has been extensively studied, but its integration into risk models has produced inconsistent results, likely due to its non-specific nature [11, 12]. Recent advancements in proteomic technologies enable the simultaneous measurement of multiple inflammation-related proteins, offering a more comprehensive assessment of the inflammatory processes underlying cardiovascular risk [13]. Large-scale studies have demonstrated the potential of proteomic biomarkers to improve cardiovascular risk prediction [14–17]. However, most of the previous studies lacked external validation sets and they did not address the specific value of inflammation-related proteins measured by multiplex technologies in MACE prediction.

This study aims to address this gap in the literature by evaluating in large-scale data from two European cohorts whether the incorporation of inflammation-related proteins into the SCORE2 model enhances 10-year MACE risk prediction, using a sex-specific selection approach.

## Methods

### Study population

The UK Biobank (UKB) is a large prospective cohort study with 502,414 participants, aged 37 to 73 years, recruited from 13 March 2006 to 1 October 2010 across 22 assessment sites in England, Scotland, and Wales [18]. The UK Biobank Pharma Proteomics Project (UKB-PPP) is a collaboration with 13 biopharmaceutical companies, generated proteomic data from 54,219 participants, including 46,595 randomly selected from the baseline cohort, 6,376 selected for 122 specific diseases, and 1,268 from a COVID-19 study [13].

The ESTHER study is an ongoing population-based cohort study with 9,940 participants, aged 50 to 75 years, recruited between July 2000 and June 2002 in Saarland, a federal state in South-West Germany. The recruitment took place during standard health checkups by general practitioners (GPs). Follow-ups were conducted every two to three years thereafter [19]. Among these participants, proteomic data were measured for 8,798 participants.

Detailed inclusion and exclusion criteria are provided in Supplemental Methods 1 and illustrated in Supplemental Figure S1. Following the application of these criteria, the final analytic sample included 47,382 participants from the UKB and 6,280 participants from the ESTHER study.

### Measurement of CRP and inflammation-related proteins

CRP was measured using immunoturbidimetric analysis with a Beckman Coulter AU5800 in UKB and an ADVIA 2400 in ESTHER.

In the UKB, proteomic profiling was performed on EDTA plasma samples collected at baseline. The Olink Explore 3072 platform (Olink Proteomics, Uppsala, Sweden) was used, which allowed the detection of 2,923 unique proteins. Measurements are reported as Normalised Protein eXpression (NPX) units. Detailed methods on the assay, sample selection, and handling have been described previously [13]. In the ESTHER study, levels of 92 inflammation-related proteins were measured in baseline serum samples with the Olink Target 96 inflammation panel [20, 21]. These 92 proteins are also part of the Olink Explore 3072 panel applied in the UKB. Overall, 19 proteins from the inflammation panel were excluded after applying the following exclusion criteria: Proteins with more than 20% missing values or with over 25% of their values falling below the limit of detection. This left *n* = 73 proteins for analysis, and the list of the protein names is shown in Supplemental Table S1. Further details on the proteomics measurements in the two studies can be found in Supplemental Methods 2.

### Variables of the SCORE2 model

The SCORE2 model, designed for adults without diabetes aged 40 to 69, includes age, sex, high-density lipoprotein cholesterol (HDL-C), total cholesterol, systolic blood pressure (SBP), and smoking status [4].

For both cohorts, standardized questionnaires were utilized to collect data on age, sex, and smoking status (current or not). HDL-C and total cholesterol were measured with an enzymatic method in both cohorts (UKB: Beckman Coulter AU5800; ESTHER: Cobas 8000 C701). SBP measurements were conducted by automated reading of the Omron device at the left upper arm in the UKB. In the ESTHER study, SBP measurement methods varied by the GPs and the results were documented in the physician’s medical conditions report of the health check-up [22].

### Outcome ascertainment

The primary endpoint was MACE, defined as a composite of cardiovascular death, non-fatal myocardial infarctions, and non-fatal strokes, with a detailed definition provided in Supplemental Table S2 and Supplemental Methods 3. This was aligned with the criteria of the SCORE2 model, except for a broader classification of non-fatal strokes in the ESTHER study due to the inability to differentiate stroke subtypes in this cohort. Non-cardiovascular deaths were considered as competing events in the analysis. Participants were followed from baseline until the first occurrence of a MACE event, death, or the end of the ten-year follow-up period, whichever occurred first.

### Statistical analyses

#### General remarks

All analyses were performed using R software (version 4.3.0, R Foundation for Statistical Computing, Vienna, Austria). Statistical significance was defined as *P*-values < 0.05 for two-sided tests. Missing values of variables of the SCORE2 model (HDL-C had the highest proportion of missing values in both cohorts with 13.1% in UKB and 2.8% in ESTHER) and proteins (mostly complete with a few proteins with up to 20% of missing values) were single imputed using the chained equations method with random forest algorithms implemented in the R package *miceRanger* (version 1.5.0).

#### Biomarker selection and model derivation

**Fig. 1:**
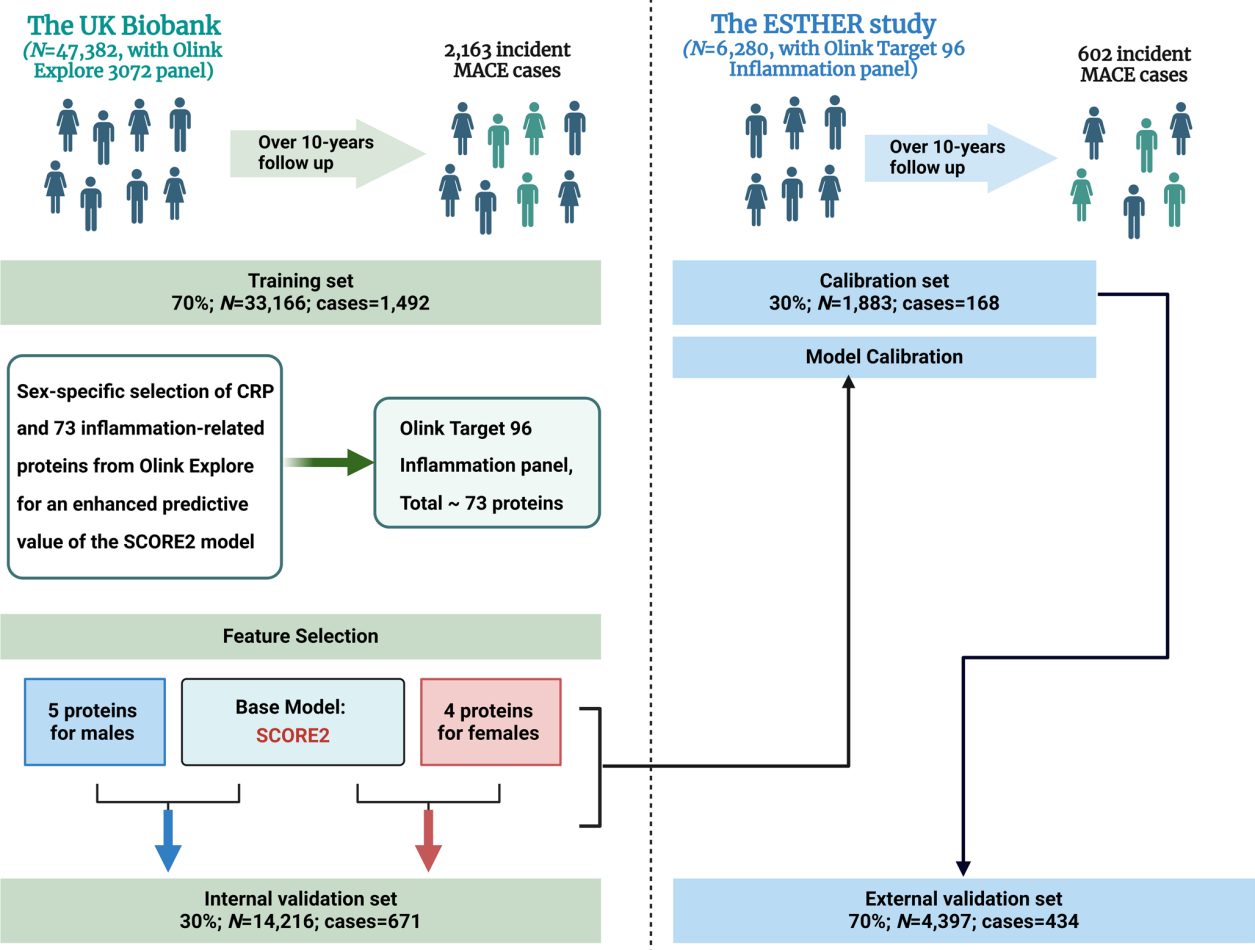
Study design. *MACE* Major adverse cardiovascular events

Figure 1 gives an overview on the study outline. The UKB dataset was randomly split into a derivation set (70%) and a validation set (30%) for both male and female participants separately. Protein selection was conducted independently within each sex stratum to develop sex-specific risk algorithms.

Feature selection among CRP and 73 inflammation-based proteins was performed using the Least Absolute Shrinkage and Selection Operator (LASSO) regression [23]. A nested ten-fold cross-validation was conducted within 200 bootstrap samples. For each bootstrap sample, ten-fold cross-validation was used to optimize the regularization parameter λ by minimizing the validation error. The biomarkers of interest were ranked based on their selection frequency across the 200 bootstrap iterations. Biomarkers selected in at least 95% of the bootstrap samples (score ≥ 190) were designated as proteins of interest. This high threshold was chosen to enhance model generalizability and minimize overfitting, ensuring that only the most robust predictors were included [24]. The selected inflammation-related biomarkers were then incorporated into the SCORE2 model to develop new sex-specific risk algorithms. To account for the competing risk of non-cardiovascular death, model derivation was conducted using Fine and Gray subdistribution hazard regression as implemented in the *cmprsk* R package (version 2.2–11) [25]. Additionally, we used Spearman correlation analysis to investigate the correlations between CRP and the selected proteins in both male and female participants.

#### Model performance validation

Internal validation of new sex-specific risk algorithms was performed in 30% of the UKB set (Fig. 1). As the relative NPX values of the OLINK Explore and OLINK Target panels are not comparable, the UKB training set coefficients could not be directly applied to the ESTHER cohort. Therefore, the ESTHER cohort was randomly split into a calibration set (30%) and a validation set (70%) and the calibration set was used to obtain new ß coefficients for the selected proteins from the OLINK Inflammation 96 Target panel and the other variables of the SCORE2 risk score.

To ensure fair comparison between models, we re-estimated the SCORE2 coefficients in each derivation set. Model performance was evaluated using Harrell’s C-index, accounting for competing risks with the *riskRegression* R package (version 2023.03.2) and the receiver operating characteristic (ROC) curve. The statistical significance of improvements in the C-index was evaluated using the method for comparing correlated C-indices in survival analysis proposed by Kang et al. implemented in the R package *compareC* (version 1.3.2) [26]. Risk reclassification was evaluated using the net reclassification index (NRI) and the integrated discrimination index (IDI) [27]. For the UKB, classified as a low-risk region in the SCORE2 model, we calculated the NRI using pre-specified 10-year MACE risk categories of 0–5%, > 5–10%, and > 10%. For the ESTHER study, considered a moderate-risk region, we used categories of 0–7.5%, > 7.5–15%, and > 15%. Model calibration was assessed by plotting observed MACE event rates against predicted rates across deciles of absolute predicted risk. Furthermore, the incremental contribution of each protein to discrimination was evaluated based on the increase in the C-index.

#### Associations of selected proteins with MACE

To report hazard ratios (HRs) and 95% confidence intervals (CIs) for the selected proteins (per one standard deviation (SD) increment) associated with 10-year MACE incidence in male and female participants, the proteins were individually added to Cox proportional hazards regression models in the validation set. These models were adjusted for SCORE2 model variables using the *survival* package (version 3.5-5) in R.

#### Sensitivity analyses

Several sensitivity analyses were conducted to assess the robustness of the findings. First, all primary analyses were repeated after excluding 5,114 UKB participants and 258 ESTHER participants who were not randomly selected for proteomics measurements.

Second, to compare the predictive contribution of inflammation-related proteins with the one of established cardiac-specific biomarkers NT-proBNP (N-terminal pro-brain natriuretic peptide) and cTnI (cardiac troponin I), we evaluated two further prediction models in the internal validation set: the refitted SCORE2 model plus NT-proBNP and cTnI, and a combined model including the SCORE2 variables along with both the cardiac and inflammation-related biomarkers.

Third, we examined whether statin use had an impact on the results in the internal validation set because statins could reduce subclinical inflammation [28]. Statin use was recorded by study personal of the UKB at baseline by examining the medication brought to the assessment center by the study participants. We first assessed whether statin users had altered protein levels compared to non-users by a linear regression model with log-transformed biomarkers (to ensure normal distribution) and adjustment for the variables of the SCORE2 model. Second, the performance of protein-extended model was compared to the refitted SCORE2 model in the internal validation set separately among statin users and non-users.

## Results

### Baseline characteristics and MACE case numbers

Table 1 summarizes the baseline characteristics of the 47,382 UKB participants and 6,280 ESTHER participants included in the analysis. The average age of UKB participants was 56.4 years (SD 8.2), with 44.1% being male. Over the 10-year follow-up period, 2,163 MACE and 1,713 competing non-cardiovascular deaths were recorded. In the ESTHER study, the mean age was 61.7 years (SD 6.6), and 41.9% of participants were male, with a total of 602 MACE and 399 competing non-CVD deaths documented during the same follow-up period.

**Table 1:** Baseline characteristics of the analyzed individuals from the UK biobank and the ESTHER study

Baseline characteristics	UK Biobank (*N* = 47,382)	ESTHER (*N* = 6,280)
Male sex, N (%)	20,900 (44.1)	2,634 (41.9)
Age (years), mean (SD)	56.4 (8.2)	61.7 (6.6)
Current smoker, N (%)	4,909 (10.4)	1,030 (16.4)
Systolic blood pressure (mmHg), mean (SD)	139.5 (19.8)	139.2 (19.1)
Total cholesterol (mmol/L), mean (SD)	5.8 (1.1)	5.9 (1.2)
HDL cholesterol (mmol/L), mean (SD)	1.5 (0.4)	1.4 (0.4)

*HDL* High-density lipoprotein; *SD* Standard deviation.

### Biomarker selection and model derivation

A total of 7 proteins were selected to enhance MACE risk prediction in the SCORE2 model within the derivation cohort using LASSO analysis and bootstrapping. CRP was not selected as a predictor in the final models. Among the 7 selected proteins, 2 were identified in both sexes, 3 were specific for males, and 2 were specific for females.

**Fig. 2:**
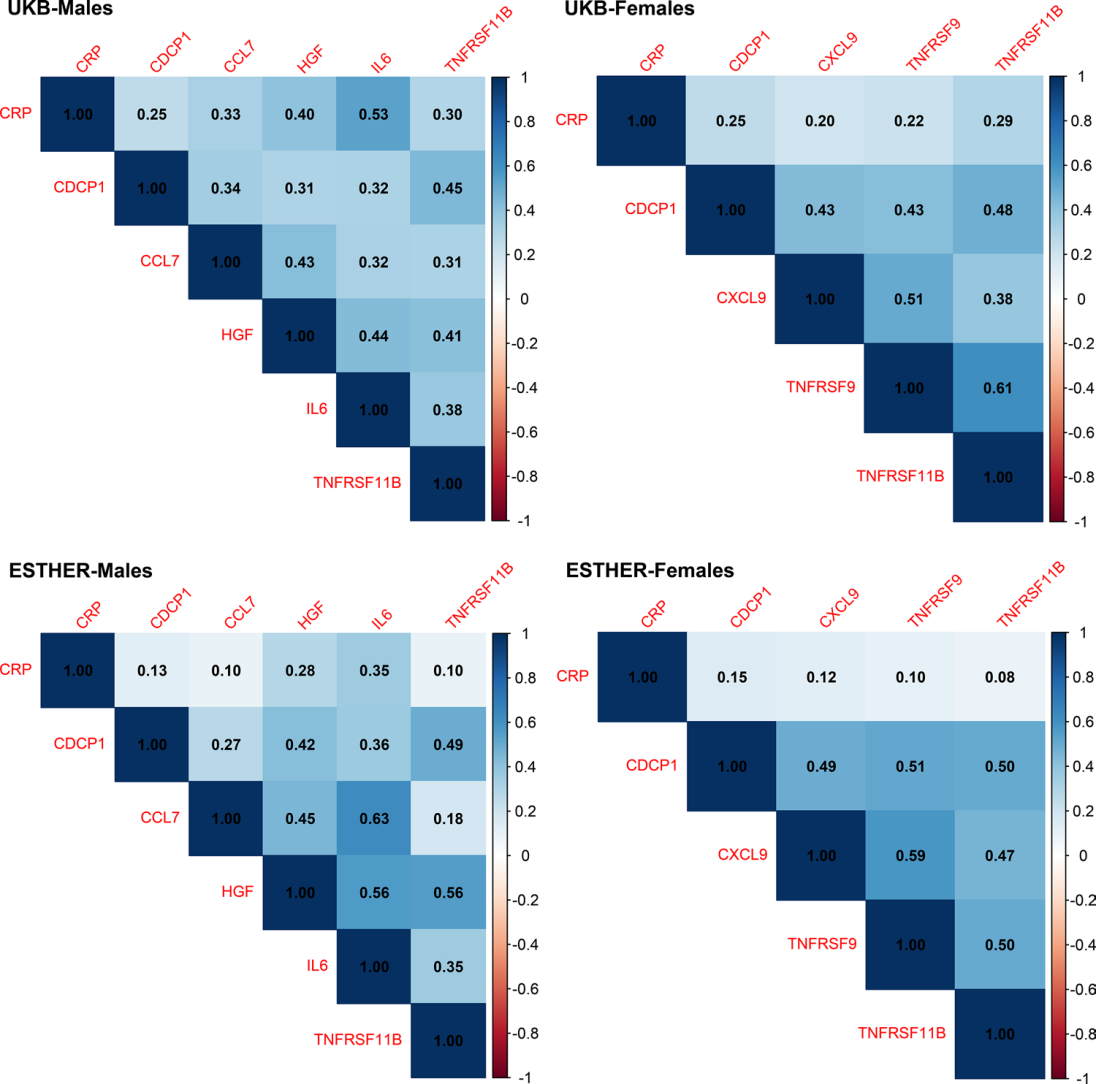
Spearman correlation coefficients of C-reactive protein and the selected inflammation-related proteins stratified by sex in the UK Biobank and ESTHER study

Figure 2 presents the Spearman correlation coefficient (r) matrices for CRP and the 5 selected inflammation-related proteins in males and the 4 selected ones in females. Most of the selected proteins were moderately correlated with each other and with CRP with correlation coefficients in the range from *r* = 0.2–0.49 with a few exceptions of high correlations with correlation coefficients between *r* = 0.5 and *r* = 0.62. While correlation of all selected proteins with CRP was low among females (all *r* ≤ 0.29), there was a high correlation between CRP and IL6 (Interleukin-6) among males (*r* = 0.53).

### Associations of selected proteins with MACE

Supplemental Figure S2 provides HRs with 95% CIs for the associations between each selected protein and MACE in males and females in both cohorts. In the UKB, all selected proteins showed significant positive associations with MACE across sexes. However, none of these associations were observed in the ESTHER study. The observed differences may be partially explained by the fasting status of participants, with non-fasting samples comprising 95.7% of the UKB but only 10.7% of the ESTHER cohort. Supplemental Table S3 highlights substantial differences in mean protein concentrations for most of the selected proteins between fasting and non-fasting participants in the ESTHER study.

### Model performance validation

**Fig. 3:**
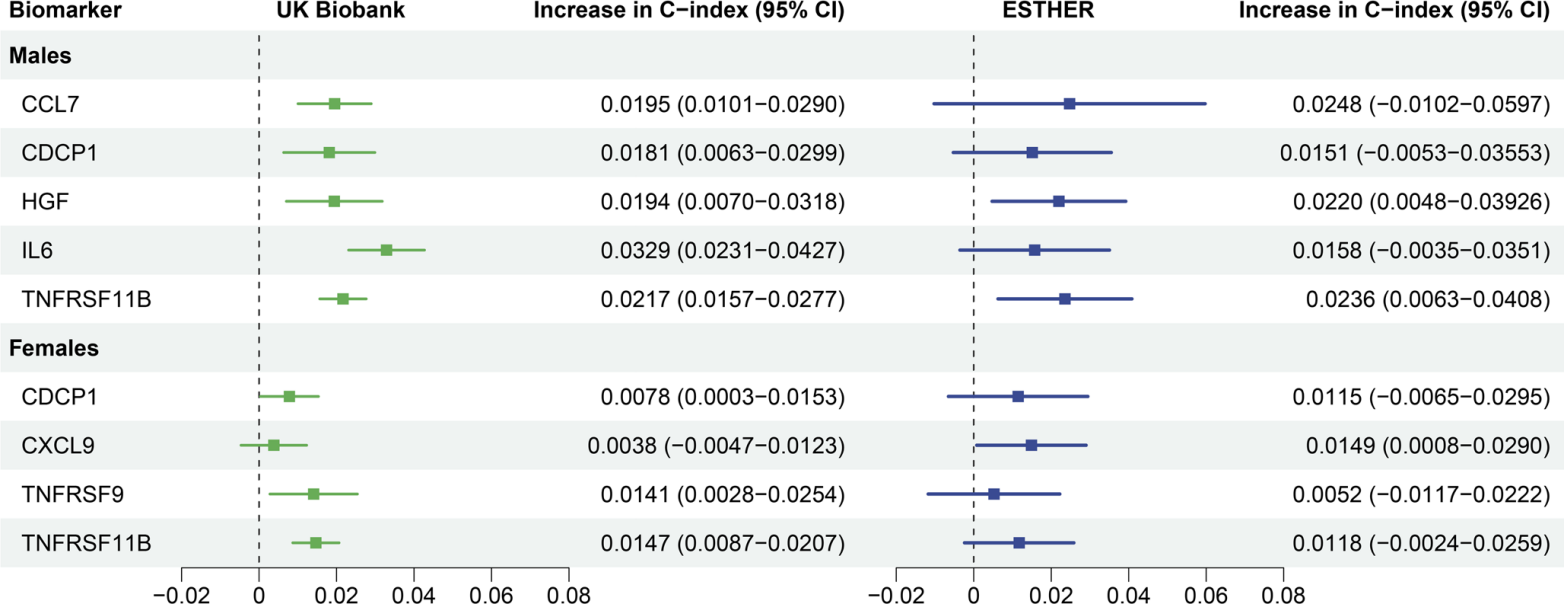
The incremental discrimination of each protein for the model after the selected proteins were added to SCORE2 separately in different sexes in the internal validation (30% of UK Biobank, *N* = 14,216) and external validation (70% of ESTHER, *N* = 4,397). *CDCP1* CUB domain-containing protein 1, *CCL7* C-C motif chemokine 7, *CXCL9* C-X-C motif chemokine 9, *HGF* Hepatocyte growth factor, *IL6* Interleukin-6, *TNFRSF9* Tumor necrosis factor receptor superfamily member 9, *TNFRSF11* Tumor necrosis factor receptor superfamily member 11.

Despite differences in the HRs, the predictive value of the selected proteins observed in the UKB was validated in the ESTHER study. Figure 3 illustrates the incremental contributions of each protein to the C-index in the internal set (UKB) and external validation set (ESTHER). Among males, all selected proteins improved discrimination significantly in the UKB. In the ESTHER study, the increases in the C-index were of similar magnitude but only HGF (Hepatocyte growth factor) and TNFRSF11B (Tumor necrosis factor receptor superfamily member11B) reached statistical significance. For females, all selected proteins except CXCL9 (C-X-C motif chemokine 9) significantly improved the C-index in the UKB. In contrast, CXCL9 was the only protein with a statistically significant C-index improvement among female participants of the ESTHER study. Overall, the results in UKB and ESTHER were comparable for females as well.

**Fig. 4:**
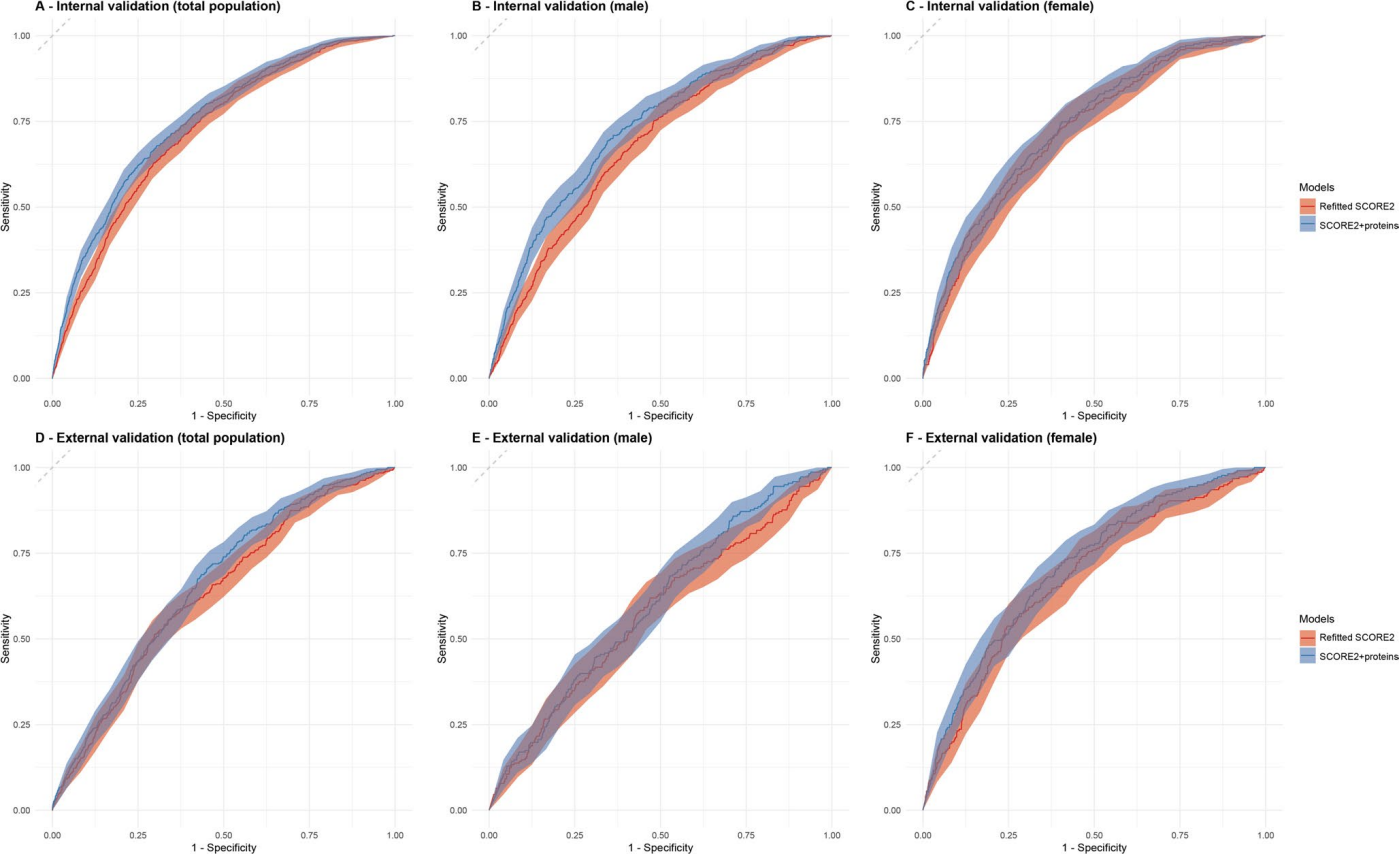
ROC curves of the SCORE2 model with and without proteomics data for 10-year MACE risk prediction in the internal validation (30% of UK Biobank, *N* = 14,216) and external validation (70% of ESTHER, *N* = 4,397). The ROC curves for the refitted SCORE2 model (red line) and the SCORE2 model augmented with proteomic biomarkers (blue line) for predicting 10-year major adverse cardiovascular events (MACE). The results are shown for the total population of UKB (**Panel A**), males of UKB (**Panel B**), females of UKB (**Panel C**), total population of ESTHER study (**Panel D**), males of ESTHER study (**Panel E**), females of ESTHER study (**Panel F**)

Supplemental Tables S4 and S5 show the ß coefficients of the variables of the final prediction model for males and females (SCORE2 variables extended by all selected proteins) in the UK Biobank and ESTHER study, respectively. Table 2 presents the predictive performance metrics of the original SCORE2 model, the refitted SCORE2 model, and the protein-extended models. In the derivation set, integrating inflammation-related proteins increased the C-index by 0.030 in the total population, with improvements observed in both sexes (+ 0.044 in males and + 0.022 in females). Similar improvements were seen in the internal validation set (+ 0.034, + 0.050, and + 0.022 in the total population, males, and females, respectively. External validation in the ESTHER study confirmed the enhanced performance, with the C-index increasing by 0.029 in the total population and by 0.033 in both males and females (Fig. 4).

**Table 2:** Metrics of the predictive performance of the SCORE2 model for 10-year MACE risk without and with extension by proteins

Metrics	Males ^a^	Females ^b^	Overall
Derivation set (70% of UK Biobank)			
Total sample size (*N* = 33,166) / MACE case number (*N* = 1,492)			
C-Statistics (SCORE2)	0.678 (0.662, 0.695)	0.717 (0.698, 0.737)	0.712 (0.700, 0.724)
C-Statistics (Refitted SCORE2)	0.682 (0.666, 0.699)	0.718 (0.699, 0.738)	0.713 (0.701, 0.725)
C-Statistics (+ Proteins)	0.726 (0.710, 0.742)	0.740 (0.721, 0.759)	0.743 (0.732, 0.755)
*P*-values for C-Statistics comparisons^c^	**< 0.001**	**< 0.001**	**< 0.001**

Internal validation set (30% of UK Biobank)			
Total sample size (*N* = 14,216) / MACE case number (*N* = 671)			
C-Statistics (SCORE2)	0.683 (0.658, 0.707)	0.713 (0.683, 0.743)	0.711 (0.693, 0.729)
C-Statistics (Refitted SCORE2)	0.684 (0.659, 0.709)	0.720 (0.690, 0.751)	0.716 (0.698, 0.734)
C-Statistics (+ Proteins)	0.734 (0.710, 0.759)	0.742 (0.712, 0.772)	0.750 (0.732, 0.768)
*P*-values for C-Statistics comparisons^c^	**< 0.001**	**0.001**	**< 0.001**
NRI categorical total (%)^d^	12.8 (-6.8, 23.1)	**8.9 (1.7**,** 15.5)**	**12.4 (5.2**,** 16.3)**
NRI categorical events (%)^d^	4.0 (-2.7, 10.2)	**8.5 (1.2**,** 15.2)**	6.7 (-1.8, 10.3)
NRI categorical non-events (%)^d^	8.8 (-14.2, 22.8)	0.4 (-0.3, 13.8)	**5.7 (3.6**,** 8.6)**
IDI	**0.025 (0.017**,** 0.033)**	**0.011 (0.002**,** 0.021)**	**0.019 (0.012**,** 0.028)**

External validation set (70% of ESTHER)			
Total sample size (*N* = 4,397) / MACE case number (*N* = 434)			
C-Statistics (SCORE2)	0.627 (0.589, 0.665)	0.714 (0.679, 0.749)	0.678 (0.653, 0.704)
C-Statistics (Refitted SCORE2)	0.606 (0.563, 0.649)	0.712 (0.674, 0.749)	0.674 (0.647, 0.701)
C-Statistics (+ Proteins)	0.639 (0.597, 0.682)	0.745 (0.707, 0.782)	0.703 (0.675, 0.730)
*P*-values for C-Statistics comparisons^c^	**0.023**	**< 0.001**	**< 0.001**
NRI categorical total (%)^e^	1.5 (-2.9, 6.7)	**25.6 (1.0**,** 41.3)**	3.0 (-0.6, 23.4)
NRI categorical events (%)^e^	**4.6 (0.5**,** 9.2)**	0.4 (-13.8, 9.2)	4.8 (-2.0, 8.4)
NRI categorical non-events (%)^e^	**-3.0 (-5.0**,** -1.0)**	25.1 (-2.0, 46.2)	-1.8 (-2.6, 25.4)
IDI	0.003 (-0.007, 0.014)	0.012 (-0.005, 0.031)	0.006 (-0.004, 0.014)

Numbers in bold indicate statistically significant results (P < 0.05)

*IDI* Integrated discrimination improvement; NRI, net reclassification improvement.

^a^ Proteins that were included for males were CCL7, CDCP1, HGF, IL6, and TNFRSF11B.

^b^ Proteins that were included for females were CDCP1, CXCL9, TNFRSF9, and TNFRSF11B.

^c^ P-values compare the refitted SCORE2 model with the SCORE2 model extended by proteins.

^d^ NRI calculated with the pre-specified 10-year MACE risk categories of 0–5%, > 5–10%, and > 10%.

^e^ NRI calculated with the pre-specified 10-year MACE risk categories of 0–7.5%, > 7.5–15%, and > 15%.

Integration of the selected proteins also enhanced reclassification metrics. In internal validation, the NRI for the total population was 12.4%, with notable improvements among females (NRI: 8.9%) and males (NRI: 12.8%). In external validation, the NRI in the total population was 3.0%, with a much higher NRI observed among females (25.6%) than among males (1.5%).

Calibration curves (Supplemental Figure S3) demonstrated good alignment between observed and predicted MACE rates for both the refitted SCORE2 and the model extended by proteins in the internal validation and in external validation for females. In males and the total population of the external validation set, the calibration was good for an up to 15% predicted MACE risk and poor for higher predicted risks for both the refitted SCORE2 model and the protein-extended model.

### Sensitivity analyses

We conducted several sensitivity analyses to evaluate the robustness of our findings. First, excluding non-randomly selected participants from both cohorts, demonstrated a slight improvement in the C-index for both the SCORE2 model and the proteins extended model in the total population (Supplemental Table S6). Consistent with the main analysis, the protein extended model outperformed the C-index of the refitted SCORE2 model in both the internal and external validation set.

**Fig. 5:**
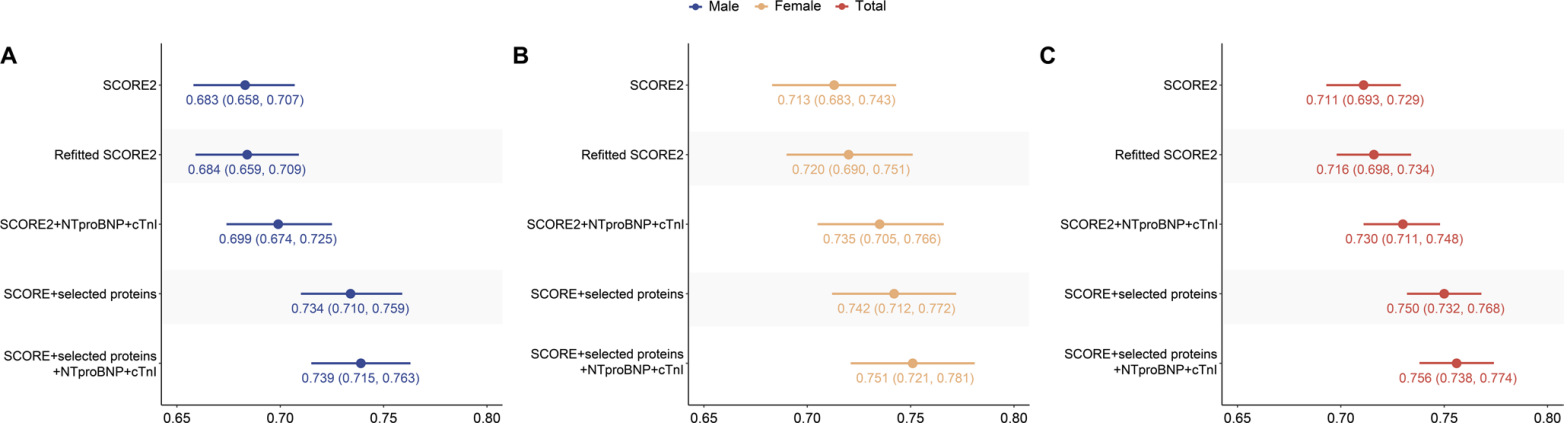
Comparison of C-index for five risk prediction models based on SCORE2 and its extensions in the internal validation (30% of UK Biobank, *N* = 14,216). C-statistics for five models are shown: (1) the original SCORE2 model; (2) the refitted SCORE2 model; (3) SCORE2 plus cardiac-specific biomarkers (NT-proBNP and cTnI); (4) SCORE2 plus the seven selected inflammation-related proteins; and (5) SCORE2 plus both cardiac-specific and inflammation-related biomarkers. The results are shown for males (**Panel A**), females (**Panel B**), and the total population (**Panel C**). *cTnI*, Cardiac troponin I; *NTproBNP*, N-terminal pro-brain natriuretic peptide

Second, we compared the added predictive value of inflammation-related proteins and cardiac-specific biomarkers (Fig. 5). Both cardiac and inflammation-based proteins statistically significantly improved the C-index of the refitted SCORE2 when added to the refitted SCORE2 (all *P* > 0.05). However, the addition of the inflammation-related proteins added more to the C-index than the cardiac biomarkers. While the model including both cardiac biomarkers and inflammation-related proteins showed the highest C-index, the difference compared to the protein-extended model alone was not statistically significant in neither men, women, nor the total population (all *P* > 0.05).

Third, we investigated whether statin use altered the results. At baseline, 11.2% of UKB participants were statin users. After adjustment for SCORE2 variables, all selected proteins except TNFRSF9 were significantly increased among statin users compared to non-users (Supplemental Table S7). While C-indices were generally lower among statin users for all models, the inflammation-related protein-extended models consistently outperformed the refitted SCORE2 models in both statin users and non-users (Supplemental Figure S4).

## Discussion

In this study involving large-scale data from the UKB and the ESTHER study, we demonstrated that incorporating selected inflammation-related proteins into the SCORE2 model significantly improves the prediction of 10-year MACE in individuals without prior CVD or diabetes. By employing a sex-specific selection approach using LASSO regression and bootstrapping, we selected 7 inflammation-related proteins, of which 2 were selected for both sexes, 3 only for males, and 2 only for females. The inclusion of these proteins led to significant increases in the C-index in both internal and external validations, as well as improvements in reclassification metrics.

### Comparison with previous studies & novelty

Inflammation is a key mechanism in the development of atherosclerosis and the triggering of major cardiovascular events [29]. CRP as a non-specific marker of inflammation, has been extensively investigated over the past two decades for its value in cardiovascular risk prediction [12]. Some studies have shown that adding CRP to risk models can improve the C-index [30–32]. For example, in a cohort of 3,435 men aged 45 to 74 years from southern Germany, adding CRP to the Framingham risk score increased the C-index from 0.735 to 0.750 [30]. However, many other studies have found that incorporating CRP does not significantly enhance the model’s discriminatory ability [12]. In our study, we included CRP in the protein selection process, but it was not selected as a predictor. This may be due to the limited additional information CRP provides compared to other inflammation-related proteins. Especially IL-6 is highly correlated with CRP (which we also observed in male participants of the UKB) and is more strongly related to MACE than CRP [33, 34]. We presume that this is the reason why IL6 and not CRP was selected for the risk prediction model for males in our derivation set.

The recent development of large-scale targeted proteomics has offered unprecedented opportunities to identify novel circulating biomarkers for MACE [35]. For instance, Helgason et al. found in an Icelandic cohort that adding a protein risk score to traditional risk models increased the C-index by 0.014 [14]. Similarly, Hoogeveen et al. included 50 proteins in a cardiovascular disease risk model in the EPIC-Norfolk cohort and ranked the importance of these proteins [15]. Notably, most of these proteins were inflammation-related, and 5 of the 7 proteins selected in our study — CXCL9, IL6, CDCP1 (CUB domain-containing protein 1), TNFRSF11B, and TNFRSF9 (Tumor necrosis factor receptor superfamily member 9) — were ranked 6th, 8th, 12th, 18th, and 23rd, respectively, in their work. Additionally, Royer et al. investigated the enhancement of the SCORE2 model’s predictive ability with proteomic biomarkers in approximately 38,000 participants from the UKB [17]. They found that integrating 114 proteins (selected from 2,919 proteins of the Olink Explore panel) into the model increased the area under the curve (AUC) by 0.031 (from 0.740 to 0.771). To our knowledge, this study is the first to focus specifically on inflammation-related proteomic biomarkers for improving MACE risk prediction. Compared to Royer et al., we selected far fewer number of proteins (7 vs. 114) and observed a similar improvement in the C-index of 0.034 (from 0.716 to 0.750) in the UKB population.

These findings highlight the important role of subclinical inflammation in the prediction of MACE, which was not detected or underestimated by previous studies, which only evaluated CRP. Measuring a small number of more specific proteins for different aspects of subclinical inflammation (similar to the 5 proteins for males and the 4 proteins for females derived from our study) may offer a practical balance between improving model accuracy and maintaining feasibility for routine clinical practice.

### Potential clinical utility

Regarding practical implementation, we acknowledge that reproducibility, availability, and costs are key considerations. All of the 5 selected proteins for males and the 4 selected ones for women are in the library of the OLINK Flex assay, which can measure up to 30 proteins with absolute concentrations. Thus, the selected proteins could be measured reproducibly and simultaneously at relatively low costs because the OLINK Flex measurements are more affordable compared to large-scale proteomics assays. The last price we got for a scientific project was 70 € per sample for the simultaneous measurement of 21 proteins with the OLINK Flex kit measurement. The availability of this technology is given because the OLINK technology can measure large sample volumes in very short time and the company has contracts with multiple labs in several countries by now that offer these measurements. However, before the developed prediction model can be used in clinical routine, the algorithm needs to be recalibrated with the proteins measured in absolute concentrations with OLINK Flex kits in cohorts with only fasting blood samples. This should ideally be done in different European countries with varying underlying cardiovascular risk, as done before for the calibration of the SCORE2 algorithm [4].

While the combination of the selected proteins with the cardiac biomarkers NT-proBNP and cTnI achieved an even higher C-index in our study, the added benefit over the “protein-only + SCORE2” model was not statistically significant in neither women, men nor the total population (all *P* > 0.05), suggesting that inflammation-based markers provide a higher predictive value than NT-proBNP and cTnI and could even replace them in a prediction model without much loss in predictive information. These findings underscore the high value of inflammation-related proteins in cardiovascular risk prediction.

While we acknowledge that inflammation-related proteins can fluctuate due to factors such as recent food intake or acute illness [36], they seem to be stable enough over time on the population-level for the long-term prediction of MACE. Otherwise they would not have been selected in our study as improvements of the SCORE2 model. Nevertheless, on the individual level, the prediction model should only be used in practice with fasting blood samples and not in patients with acute infections or acute phases of illnesses known to cause acute, clinically relevant inflammation.

### Biological roles of selected inflammatory proteins in cardiovascular risk prediction

The 7 inflammation-related proteins selected in our study — IL6, HGF, CDCP1, TNFRSF11B, CCL7 (C-C motif chemokine 7), TNFRSF9, and CXCL9 — reflect processes involved in the initiation and progression of CVD, including inflammation, immune responses, and vascular remodeling.

IL6 is a pro-inflammatory cytokine central to the acute-phase response and immune activation [37]. By promoting endothelial dysfunction and facilitating immune cell recruitment, it plays a key role in atherogenesis and the destabilization of plaques [38]. Elevated IL6 levels have consistently been associated with cardiovascular outcomes, reflecting chronic low-grade inflammation that underlies many CVD processes [39].

HGF serves as a dual mediator, promoting tissue repair and angiogenesis, while also being implicated in pathological vascular remodeling [40]. Elevated HGF levels are thought to indicate vascular injury and reparative efforts in response to endothelial damage [41].

Proteins involved in cell adhesion and apoptosis include CDCP1 and TNFRSF11B. CDCP1 contributes to vascular endothelial integrity and repair mechanisms but may also indicate ongoing endothelial damage in pathological conditions [42]. TNFRSF11B functions as a decoy receptor for RANKL and modulates vascular calcification and inflammation [43]. Elevated TNFRSF11B levels have been associated with arterial stiffness, vascular calcification, and an increased risk of cardiovascular events, potentially reflecting maladaptive remodeling in atherosclerosis [44].

Chemokines, such as CCL7, CXCL9, and TNFRSF9, reflect immune cell recruitment and inflammation in the vascular microenvironment. CCL7 recruits monocytes and macrophages, which are critical for foam cell formation and plaque development [45]. CXCL9 supports T-cell recruitment and promotes chronic inflammatory states within atherosclerotic plaques [46]. TNFRSF9 is expressed on T-cells and endothelial cells and amplifies inflammatory signaling, exacerbating immune cell infiltration and plaque instability [47].

### Strengths and limitations

The main strengths of this study are its large sample size, the robustness of the results confirmed by effective external validation, and rigorous case ascertainment. Additionally, this study is pioneering in deriving sex-specific predictive models for cardiovascular risk by selecting proteomic biomarkers separately for males and females, which reflects biological differences between sexes and supports more precise and personalized risk stratification.

However, several limitations should be acknowledged. First, the study cohorts differed in baseline cardiovascular risk, with the UK Biobank representing a low-risk population and the ESTHER study representing an intermediate-risk population. Second, the Olink platforms used in this study differed and provided cohort-specific relative NPX values, which implied that the variable coefficients derived from the UKB derivation set could not be directly applied to the ESTHER external validation set. We addressed this limitation by splitting the validation cohort into two parts, with one part used for calibration to obtain new ß-coefficients. However, this approach may have led to an overestimation of the model performance in external validation. Third, the fasting status of participants varied markedly between cohorts, with the UK Biobank predominantly comprising non-fasting participants (95.7%) compared to only 10.7% in the ESTHER study. As most of the protein concentrations also varied by fasting status, this limitation was another reason for re-calibrating the model in ESTHER study. Future studies should use absolute protein quantification and study populations with 100% fasting status to obtain coefficients that are more generalizable across populations. Finally, although this study may be applicable to low-to-intermediate risk regions in Europe, larger validation cohorts from high and very high-risk regions are required to extend the generalizability of these findings.

## Conclusion

These findings underscore the pivotal role of inflammation in cardiovascular disease pathogenesis and the potential of specific inflammation-related proteins as biomarkers for risk prediction. We showed that a small number of inflammation-related proteins can enhance risk prediction without the need for extensive proteomic profiling, making it more feasible for widespread clinical implementation. If the model demonstrates comparable performance in future external validation studies, it could serve to enhance cardiovascular prevention strategies that are currently based on the SCORE2 risk charts.

## Supplementary Information

Below is the link to the electronic supplementary material.

Supplementary Material 1

## Abbreviations

AUC: Area under the curve
CCL7: C-C Motif chemokine 7
CDCP1: CUB Domain-containing protein 1
CI: Confidence intervals
cTnI: Cardiac troponin I
CVD: Cardiovascular disease
CXCL9: C-X-C Motif chemokine 9
HDL-C: High-density lipoprotein cholesterol
HGF: Hepatocyte growth factor
HR: Hazard ratio
IDI: Integrated discrimination improvement
IL6: Interleukin-6
LASSO: Least absolute shrinkage and selection operator
MACE: Major adverse cardiovascular events
NRI: Net reclassification index
NT-proBNP: N-Terminal pro-brain natriuretic peptide
TNFRSF9: Tumor necrosis factor receptor superfamily member 9
TNFRSF11B: Tumor necrosis factor receptor superfamily member11B
SBP: Systolic blood pressure
SD: Standard deviation
UKB: UK Biobank
UKB-PPP: UK Biobank pharma proteomics project
