# Supplementary material for: Circulating inflammation-related proteome improves cardiovascular risk prediction. Results from two large European cohort studies
Source: Eur J Epidemiol. 2025 Aug 13;40(10):1191–203. doi: 10.1007/s10654-025-01285-y (PMC12660460; doi:10.1007/s10654-025-01285-y)
Supplement: Supplementary file 1 — Supplementary Material 1 [file 10654_2025_1285_MOESM1_ESM.docx]

**Supplemental Materials to**

**Circulating inflammation-related proteome improves cardiovascular risk prediction. Results from two large European cohort studies**

Table of Contents

[Supplemental Methods 1. In- and exclusion criteria 2](#_Toc15648)

[Supplemental Methods 2. Details on blood proteomics measurements 2](#_Toc11730)

[Supplemental Methods 3. Outcome ascertainment 4](#_Toc22366)

[Supplemental Table S1. Biomarkers analyzed from with Olink Target 96 Inflammation panel 5](#_Toc3205)

[Supplemental Table S2. Definition of endpoint major cardiovascular event (MACE) 7](#_Toc10652)

[Supplemental Table S3. Comparison of the standardized circulating concentration of the 7 selected proteins by fasting status in the ESTHER study 8](#_Toc26337)

[Supplemental Table S4. ß-coefficients of the variables of the SCORE2 model extended by 7 proteins for 10-year prediction of major cardiovascular events in the derivation set of the UK Biobank 9](#_Toc21222)

[Supplemental Table S5. ß-coefficients of the variables of the SCORE2 model extended by 7 proteins for 10-year prediction of major cardiovascular events in the calibration set of the ESTHER study 10](#_Toc16141)

[Supplemental Table S6. Metrics of the predictive performance of the SCORE2 model for 10-year MACE risk without and with extension by proteins in random selected participants 11](#_Toc22245)

[Supplemental Table S7. Comparison of adjusted standardized circulating protein concentrations by statin use in the UK Biobank 13](#_Toc29606)

[Supplemental Figure S1. Flow-charts for participant inclusion and exclusion 14](#_Toc7431)

[Supplemental Figure S2. Associations between selected proteins and major cardiovascular events across sexes in the internal validation (30% of UK Biobank, N=14,216) and external validation (70% of ESTHER, N=4,397) 15](#_Toc10438)

[Supplemental Figure S3. Calibration curves of the SCORE2 model with and without proteomics data for 10-year MACE risk prediction in the internal validation (30% of UK Biobank, N=14,216) and external validation (70% of ESTHER, N=4,397) 16](#_Toc8657)

[Supplemental Figure S4. Performance of the SCORE2 model and its extension with inflammation-related proteins stratified by statin use in the internal validation (30% of UK Biobank, N=14,216) 17](#_Toc6475)

[References to Supplemental Materials 18](#_Toc9404)

**Supplemental Methods 1. In- and exclusion criteria**

In the UK Biobank (UKB) and ESTHER study, proteomics measurement data were available for 54,219 and 8,798 participants, respectively. In the UKB, participants with more than 50% missing proteomics data were excluded (n=1,871). In the ESTHER study, samples with a blood sample quality control warning were removed (n=275) (see **Supplemental Figure S1** for the study flowchart). Additionally, individuals with physician-diagnosed or self-reported diabetes prior to baseline, those with potentially undiagnosed diabetes (HbA_1c_ ≥ 6.5% [48 mmol/mol] or taking glucose-lowering drugs), or those with missing diabetes data were excluded in both cohorts (UKB: n=3,032 for diagnosed diabetes, n=165 for missing data; ESTHER: n=1,282 for diagnosed diabetes, n=129 for missing data). Participants with a history of major adverse cardiovascular events (MACE) prior to baseline and those with missing MACE data were also excluded (UKB: n=1,763 for previous MACE, n=6 for missing data; ESTHER: n=490 for previous MACE, n=167 for missing data on previous MACE and n=175 lost-to-follow-up for MACE). After these exclusions, the final study population consisted of 47,382 participants in the UKB and 6,280 participants in the ESTHER study.

**Supplemental Methods 2. Details on blood proteomics measurements**

***Laboratory methods***

Olink utilizes the Proximity Extension Assay (PEA) method that targets proteins via pairs of antibodies linked to complementary oligonucleotides. Details of this method have been described elsewhere [1-3]. Briefly, when both antibodies bind to their target protein, their attached oligonucleotides hybridize and are extended by a DNA polymerase, creating a unique sequence that can be quantified. The Olink Explore platform uses next-generation sequencing (NGS) for high-throughput readout, enabling the analysis of thousands of proteins simultaneously, whereas the Olink Target platform employs quantitative PCR (qPCR) for smaller, focused panels. Detection limits are established for each protein-targeting assay on each plate, calculated from negative controls executed in triplicate. Normalized Protein eXpression (NPX) values are calculated by first normalizing the data to extension control values, then applying a log2 transformation, and finally normalizing against plate controls to correct for inter-plate variability. Samples receive a warning if NPX values from internal controls deviate by more than ±0.3 NPX from the plate median within an abundance block, or if the average assay count per sample falls below 500. Assays receive a warning if the median from triplicate negative controls deviates by more than five standard deviations (SD) from the preset values established by Olink.

***UK Biobank’s Olink assay***

The baseline blood sample collection was completed at 22 local assessment centers across the UK from March 2006 to October 2010. For each participant, blood samples were collected in EDTA tubes and then immediately centrifuged at 2,500g for 10 min at 4°C to isolate plasma. Afterward, the supernatant was divided into aliquots and stored at −80 °C as soon as possible until further processing.

As previously described [4, 5], the majority of blood samples were randomly selected from blood samples collected during the baseline visit from UKB participants, and the remaining were selected by oversampling of cases for a large range of health conditions. Samples were transported on dry ice to the Olink Analysis Service in Uppsala, Sweden and proteomic profiling was conducted using the Olink Explore 3072, capturing 2,923 unique proteins as targeted by 2,941 assays. Since certain proteins are measured by multiple assays, results from some assays may be omitted to avoid redundancy and ensure the quality of the data, following strict quality control criteria.

The Olink Target 96 Inflammation panel, which detects 92 inflammation-related proteins, is a subset of the 2,923 proteins measured by the Olink Explore 3072. In this study, we focused on these 92 inflammation-related proteins. After applying quality control measures and excluding proteins with high missingness or low detection rates, 73 proteins were included in the final analysis, as detailed in **Supplemental Table S1**.

***ESTHER’s Olink assay***

Inflammation-related proteins in serum were quantified using the Olink Target 96 Inflammation panel [6, 7]. Serum samples were collected at baseline during a routine health check (2000–2002) and stored at -80°C until analysis, which took place at five distinct time points: March 2018, December 2018, September 2020, April 2023, July 2024, and December 2024 (denoted as t1, t2, t3, t4, t5, and t6). Each serum sample was extracted from aliquots that had undergone no more than two freeze-thaw cycles before being sent on dry ice to the respective laboratories for proteomic analysis. The assays were performed in different facilities: Olink Proteomics, Uppsala, Sweden (t1 and t2), the Helmholtz Center Munich, Germany (t3 and t4), and the German Cancer Research Center (DKFZ), Heidelberg, Germany (t5 and t6). To ensure data comparability across time points, the measurements were harmonized using bridging normalization with the OlinkAnalyze R package (<https://doi.org/10.32614/CRAN.package.OlinkAnalyze>), developed by the Olink Proteomics Data Science Team.

**Supplemental Methods 3. Outcome ascertainment**

In the UKB, occurrences of non-fatal myocardial infarctions and strokes were ascertained through data linkage to primary care records, hospital episode statistics, and national registries. Dates and causes of death were established using death registries from the National Health Service (NHS) Information Centre in England and Wales, and the NHS Central Register in Scotland.

As outlined in previous studies [8], the ESTHER study participants recorded incidents of myocardial infarction and stroke through standardized surveys conducted 2, 5, 8, and 11 years after baseline. These self-reported incidents were confirmed via questionnaires sent to the participants’ GPs, with 89% of myocardial infarction and 91% of stroke cases used in this analysis verified by the GPs. Additionally, a vital status check was conducted at registration offices, and death certificates from local health authorities were obtained for deceased participants.

**Supplemental Table S1.** Biomarkers analyzed from with Olink Target 96 Inflammation panel

| **Abbreviation** | **Biomarker name** | **Included in analysis?** |
| --- | --- | --- |
| 4E-BP1 | Eukaryotic translation initiation factor 4E-binding protein 1 | Yes |
| ADA | Adenosine Deaminase | Yes |
| ARTN | Artemin | No |
| AXIN1 | Axin-1 | Yes |
| Beta-NGF | Beta-nerve growth factor | No |
| CASP-8 | Caspase-8 | Yes |
| CCL11 | Eotaxin | Yes |
| CCL19 | C-C motif chemokine 19 | Yes |
| CCL20 | C-C motif chemokine 20 | Yes |
| CCL23 | C-C motif chemokine 23 | Yes |
| CCL25 | C-C motif chemokine 25 | Yes |
| CCL28 | C-C motif chemokine 28 | Yes |
| CCL3 | C-C motif chemokine 3 | Yes |
| CCL4 | C-C motif chemokine 4 | Yes |
| CD244 | Natural killer cell receptor 2B4 | Yes |
| CD40 | CD40L receptor | Yes |
| CD5 | T-cell surface glycoprotein CD5 | Yes |
| CD6 | T cell surface glycoprotein CD6 isoform | Yes |
| CD8A | T-cell surface glycoprotein CD8 alpha chain | Yes |
| CDCP1 | CUB domain-containing protein 1 | Yes |
| CSF-1 | Macrophage colony-stimulating factor 1 | Yes |
| CST5 | Cystatin D | Yes |
| CX3CL1 | Fractalkine | Yes |
| CXCL1 | C-X-C motif chemokine 1 | Yes |
| CXCL10 | C-X-C motif chemokine 10 | Yes |
| CXCL11 | C-X-C motif chemokine 11 | Yes |
| CXCL5 | C-X-C motif chemokine 5 | Yes |
| CXCL6 | C-X-C motif chemokine 6 | Yes |
| CXCL9 | C-X-C motif chemokine 9 | Yes |
| DNER | Delta and Notch-like epidermal growth factor-related receptor | Yes |
| EN-RAGE | Protein S100-A12 | Yes |
| FGF-19 | Fibroblast growth factor 19 | Yes |
| FGF-21 | Fibroblast growth factor 21 | Yes |
| FGF-23 | Fibroblast growth factor 23 | Yes |
| FGF-5 | Fibroblast growth factor 5 | Yes |
| Flt3L | Fms-related tyrosine kinase 3 ligand | Yes |
| GDNF | Glial cell line-derived neurotrophic factor | Yes |
| HGF | Hepatocyte growth factor | Yes |
| IFN_gamma | Interferon gamma | Yes |
| IL1_alpha | Interleukin-1 alpha | No |
| IL-10 | Interleukin-10 | Yes |
| IL-10RA | Interleukin-10 receptor subunit alpha | No |
| IL-10RB | Interleukin-10 receptor subunit beta | Yes |
| IL-12B | Interleukin-12 subunit beta | Yes |
| IL13 | Interleukin-13 | No |
| IL-15RA | Interleukin-15 receptor subunit alpha | No |
| IL-17A | Interleukin-17A | No |
| IL-17C | Interleukin-17C | Yes |
| IL-18 | Interleukin-18 | Yes |
| IL-18R1 | Interleukin-18 receptor 1 | Yes |
| IL2 | Interleukin-2 | No |
| IL20 | Interleukin-20 | No |
| IL-20RA | Interleukin-20 receptor subunit alpha | No |
| IL22-RA1 | Interleukin-22 receptor subunit alpha-1 | Yes |
| IL24 | Interleukin-24 | No |
| IL2RB | Interleukin-2 receptor subunit beta | No |
| IL33 | Interleukin-33 | No |
| IL4 | Interleukin-4 | No |
| IL-5 | Interleukin-5 | No |
| IL-6 | Interleukin-6 | Yes |
| IL-7 | Interleukin-7 | Yes |
| IL-8 | Interleukin-8 | Yes |
| LAP TGF-beta-1 | Latency-associated peptide transforming growth factor beta-1 | Yes |
| LIF | Leukemia inhibitory factor | No |
| LIFR | Leukemia inhibitory factor receptor | Yes |
| MCP-1 | Monocyte chemotactic protein 1 | Yes |
| MCP-2 | Monocyte chemotactic protein 2 | Yes |
| MCP-3 | Monocyte chemotactic protein 3 | Yes |
| MCP-4 | Monocyte chemotactic protein 4 | Yes |
| MMP-1 | Matrix metalloproteinase-1 | Yes |
| MMP-10 | Matrix metalloproteinase-10 | Yes |
| NRTN | Neurturin | No |
| NT-3 | Neurotrophin-3 | Yes |
| OPG | Osteoprotegerin | Yes |
| OSM | Oncostatin-M | Yes |
| PD-L1 | Programmed cell death 1 ligand 1 | Yes |
| SCF | Stem cell factor | Yes |
| SIRT2 | SIR2-like protein 2 | Yes |
| SLAMF1 | Signaling lymphocytic activation molecule | No |
| ST1A1 | Sulfotransferase 1A1 | Yes |
| STAMBP | STAM-binding protein | Yes |
| TGF-alpha | Transforming growth factor alpha | Yes |
| TNF | Tumor necrosis factor | Yes |
| TNFB | TNF-beta | Yes |
| TNFRSF9 | Tumor necrosis factor receptor superfamily member 9 | Yes |
| TNFSF14 | Tumor necrosis factor ligand superfamily member 14 | Yes |
| TRAIL | TNF-related apoptosis-inducing ligand | Yes |
| TRANCE | TNF-related activation-induced cytokine | Yes |
| TSLP | Thymic stromal lymphopoietin | No |
| TWEAK | Tumor necrosis factor (Ligand) superfamily, member 12 | Yes |
| uPA | Urokinase-type plasminogen activator | Yes |
| VEGF-A | Vascular endothelial growth factor-A | Yes |

**Supplemental Table S2.** Definition of endpoint major cardiovascular event (MACE)

| **Fatal MACE – cause-specific mortality due to any of the following:** | |
| --- | --- |
| *Endpoints included* | *ICD10-codes* |
| Hypertensive disease | I10-16 |
| Ischemic heart disease | I20-25 |
| Arrhythmias, heart failure | I46-52 |
| Cerebrovascular disease | I60-69 |
| Atherosclerosis/aortic aneurysm | I70-73 |
| Sudden death and death within 24 hours of symptom onset | R96.0-96.1 |
|  |  |
| *Endpoints excluded from the above endpoint:* | *ICD10-codes* |
| Myocarditis, unspecified | I51.4 |
| Subarachnoid haemorrhage | I60 |
| Subdural hemorrhage | I62 |
| Cerebral aneurysm | I67.1 |
| Cerebral arteritis | I68.2 |
| Moyamoya | I67.5 |
|  |  |
| **Non-fatal MACE** | *ICD10-codes* |
| Non-fatal myocardial infarction | I21-I23 |
| Non-fatal stroke | I60-69* |

***** In UK Biobank: I61, I63-I66, I69.

**Supplemental Table S3.** Comparison of the standardized circulating concentration of the 7 selected proteins by fasting status in the ESTHER study

| **Proteins (NPX)** | **Fasting**  **(N=5,413)** | **Non-fasting**  **(N=648)** | **ANOVA**  ***p*-value** |
| --- | --- | --- | --- |
|  | **Mean (95%CI)** | **Mean (95%CI)** |  |
| CDCP1 | 0.060 (0.038, 0.082) | 0.126 (0.059, 0.193) | 0.055 |
| CCL7 | 1.037 (0.962, 1.112) | 1.356 (1.117, 1.559) | 0.007 |
| CXCL9 | 0.062 (0.039, 0.086) | 0.048 (-0.017, 0.114) | 0.709 |
| HGF | -0.040 (-0.057, -0.023) | 0.031 (-0.017, 0.080) | 0.007 |
| IL6 | 0.457 (0.408, 0.506) | 0.649 ( 0.499, 0.799) | 0.012 |
| TNFRSF9 | -0.004 (-0.019, 0.011) | -0.047 ( -0.092, -0.003) | 0.068 |
| TNFRSF11B | -0.029 (-0.043, -0.015) | -0.048 (-0.089, -0.008) | 0.381 |

**Abbreviations:** CDCP1, CUB domain-containing protein 1; CCL7, C-C motif chemokine 7; CXCL9, C-X-C motif chemokine 9; HGF, Hepatocyte growth factor; IL6, Interleukin-6; NPX, Normalized Protein eXpresstion;TNFRSF9, Tumor necrosis factor receptor superfamily member 9; TNFRSF11B, Tumor necrosis factor receptor superfamily member 11B.

# **Supplemental** **Table S4.** ß-coefficients of the variables of the SCORE2 model extended by 7 proteins for 10-year prediction of major cardiovascular events in the derivation set of the UK Biobank

| Risk factor (units) | ß coefficients | |
| --- | --- | --- |
|  | Male | Female |
| **SCORE2 variables** |  |  |
| Age (per 5 years) | 0.3741 | 0.4126 |
| Current smoking | 0.6407 | 0.9844 |
| Systolic blood pressure (per 20mmHg) | 0.1117 | 0.2526 |
| Total cholesterol (per 1 mmol/L) | -0.0008 | -0.0502 |
| HDL cholesterol (per 0.5 mmol/L) | -0.0722 | -0.1139 |
| Smoking interaction with age | -0.0485 | -0.0578 |
| SBP interaction with age | -0.0820 | -0.0399 |
| Total cholesterol interaction with age | -0.0452 | -0.0286 |
| HDL interaction with age | -0.0434 | 0.0379 |
| **Additional proteins** **(per 1 SD)** |  |  |
| CDCP1 | 0.2192 | 0.1276 |
| CCL7 | 0.2023 | - |
| CXCL9 | - | 0.1639 |
| HGF | 0.3362 | - |
| IL6 | 0.1542 | - |
| TNFRSF9 | - | 0.2343 |
| TNFRSF11B | 0.2067 | 0.2531 |

**Abbreviations:** CDCP1, CUB domain-containing protein 1; CCL7, C-C motif chemokine 7; CXCL9, C-X-C motif chemokine 9; HDL, High density lipoprotein; HGF, Hepatocyte growth factor; IL6, Interleukin-6; NPX, Normalized Protein eXpresstion; SD, standard deviation; TNFRSF9, Tumor necrosis factor receptor superfamily member 9; TNFRSF11B, Tumor necrosis factor receptor superfamily member 11B.

# **Supplemental Table S5.** ß-coefficients of the variables of the SCORE2 model extended by 7 proteins for 10-year prediction of major cardiovascular events in the calibration set of the ESTHER study

| Risk factor (units) | ß coefficients | |
| --- | --- | --- |
|  | Male | Female |
| **SCORE2 variables** |  |  |
| Age (per 5 years) | 0.2364 | 0.5314 |
| Current smoking | 0.7539 | 0.9767 |
| Systolic blood pressure (per 20mmHg) | 0.1431 | 0.2696 |
| Total cholesterol (per 1 mmol/L) | 0.1087 | 0.0164 |
| HDL cholesterol (per 0.5 mmol/L) | -0.1201 | -0.1987 |
| Smoking interaction with age | -0.1960 | -0.0489 |
| SBP interaction with age | 0.0459 | -0.0255 |
| Total cholesterol interaction with age | -0.0587 | -0.0259 |
| HDL interaction with age | 0.0380 | -0.0237 |
| **Additional proteins** **(per 1 SD)** |  |  |
| CDCP1 | 0.0565 | 0.2127 |
| CCL7 | -0.0029 | - |
| CXCL9 | - | 0.0838 |
| HGF | -0.1677 | - |
| IL6 | 0.0311 | - |
| TNFRSF9 | - | 0.0202 |
| TNFRSF11B | -0.1314 | -0.3630 |

**Abbreviations:** CDCP1, CUB domain-containing protein 1; CCL7, C-C motif chemokine 7; CXCL9, C-X-C motif chemokine 9; HDL, High density lipoprotein; HGF, Hepatocyte growth factor; IL6, Interleukin-6; NPX, Normalized Protein eXpresstion; SD, standard deviation; TNFRSF9, Tumor necrosis factor receptor superfamily member 9; TNFRSF11B, Tumor necrosis factor receptor superfamily member 10B.

**Supplemental Table S6.** Metrics of the predictive performance of the SCORE2 model for 10-year MACE risk without and with extension by proteins in random selected participants

| **Metrics** | **Male** **^a^** | **Female** **^b^** | **Overall** |
| --- | --- | --- | --- |
| **Derivation set (70% of UK Biobank)** | | | |
| **Total sample size (N=29,585) / MACE case number (N=1,164)** | | | |
| C-Statistics (SCORE2) | 0.673 (0.655,0.691) | 0.724 (0.702,0.747) | 0.725 (0.711,0.738) |
| C-Statistics (Refitted SCORE2) | 0.676 (0.658,0.694) | 0.726 (0.704,0.749) | 0.727 (0.713,0.740) |
| C-Statistics (+Proteins) | 0.715 (0.698,0.733) | 0.742 (0.720, 0.764) | 0.750 (0.737,0.763) |
| *P*-values, C-Statistics comparisons^c^ | **<0.001** | **0.001** | **<0.001** |
| **Internal validation set (30% of UK Biobank)** | | | |
| **Total sample size (N=12,683) / MACE case number (N=515)** | | | |
| C-Statistics (SCORE2) | 0.675 (0.647, 0.703) | 0.710 (0.675, 0.766) | 0.722 (0.701, 0.743) |
| C-Statistics (Refitted SCORE2) | 0.677 (0.649, 0.705) | 0.713 (0.678, 0.769) | 0.724 (0.703, 0.745) |
| C-Statistics (+Proteins) | 0.725 (0.698,0.752) | 0.730 (0.696,0.764) | 0.751 (0.730,0.771) |
| *P*-values, C-Statistics comparisons^c^ | **<0.001** | **0.027** | **<0.001** |
| NRI categorical total (%)^d^ | **14.8 (2.6, 22.2)** | **14.9 (6.0, 22.8)** | **8.9 (2.6, 15.6)** |
| NRI categorical events (%)^d^ | -0.9 (-7.8, 7.3) | **16.9 (7.6, 24.4)** | 6.2 (-0.8, 13.3) |
| NRI categorical non-events (%)^d^ | 15.7 (-1.6, 22.0) | **-2.0 (-2.9, -1.3)** | **2.7 (1.2, 4.4)** |
| IDI | **0.016 (0.006, 0.028)** | **0.006 (0.000, 0.015)** | **0.014 (0.007, 0.021)** |
| **External validation set (70% of ESTHER)** | | | |
| **Total sample size (N=4,209) / MACE case number (N=387)** | | | |
| C-Statistics (SCORE2) | 0.622 (0.578, 0.665) | 0.728 (0.690, 0.767) | 0.689 (0.662, 0.716) |
| C-Statistics (Refitted SCORE2) | 0.616 (0.570, 0.661) | 0.726 (0.687, 0.766) | 0.686 (0.658, 0.714) |
| C-Statistics (+Proteins) | 0.645 (0.599,0.691) | 0.751 (0.712,0.790) | 0.709 (0.681,0.737) |
| *P*-values, C-Statistics comparisons^c^ | **0.044** | **0.009** | **<0.001** |
| NRI categorical total (%)^e^ | 3.0 (-2.2, 9.1) | **28.6 (0.6, 44.4)** | **11.2 (1.5, 36.1)** |
| NRI categorical events (%)^e^ | **6.3 (0.1, 11.4)** | -14.7 (-21.1, 6.6) | 4.9 (-11.6, 10.3) |
| NRI categorical non-events (%)^e^ | **-3.3 (-6.4, -1.3)** | 43.3 (-1.6, 57.9) | 6.3 (-3.1, 43.9) |
| IDI | 0.003 (-0.007, 0.013) | **0.015 (0.004, 0.029)** | 0.008 (-0.002, 0.016) |

**Abbreviations:** IDI, integrated discrimination improvement; NRI, net reclassification improvement.

^a^ Proteins that were included for male were CCL7, CDCP1, HGF, IL6, and TNFRSF11B.

^b^ Proteins that were included for female were CDCP1, CXCL9, TNFRSF9, and TNFRSF11B.

^c^ P-values compare the refitted SCORE2 model with the SCORE2 model extended by proteins.

^d^ NRI calculated with the pre-specified 10-year MACE risk categories of 0–5%, >5–10%, and >10%.

^e^ NRI calculated with the pre-specified 10-year MACE risk categories of 0–7.5%, >7.5–15%, and >15%.

**Supplemental Table S7.** Comparison of adjusted standardized circulating protein concentrations by statin use in the UK Biobank

| **Proteins (NPX)** | **Statin users**  **(N=5,296)** | **Non-statin users**  **(N=42,086)** | **β (95% CI)^a^, *p*-value** |
| --- | --- | --- | --- |
|  | **Mean (95%CI)** | **Mean (95%CI)** |  |
| CDCP1 | 0.113 (0.096, 0.130) | 0.093 (0.084, 0.102) | 0.020 (0.003, 0.037), 0.020 |
| CCL7 | 0.154 (0.134, 0.173) | 0.076 (0.066, 0.086) | 0.077 (0.058, 0.097), <0.001 |
| CXCL9 | 0.081 (0.059, 0.103) | 0.033 (0.022, 0.044) | 0.048 (0.026, 0.069), <0.001 |
| HGF | 0.135 (0.123, 0.147) | 0.072 (0.066, 0.078) | 0.063 (0.051, 0.074), <0.001 |
| IL6 | 0.203 (0.178, 0.229) | 0.159 (0.147, 0.172) | 0.044 (0.019, 0.069), <0.001 |
| TNFRSF9 | 0.060 (0.047, 0.073) | 0.050 (0.043, 0.057) | 0.010 (-0.003, 0.023), 0.148 |
| TNFRSF11B | 0.166 (0.152, 0.179) | 0.109 (0.102, 0.116) | 0.010 (0.000, 0.019), 0.044 |

^a^ Linear regression estimate adjusted for age, sex, systolic blood pressure, total cholesterol, HDL cholesterol, and smoking status (SCORE2 variables).

**Abbreviations:** CDCP1, CUB domain-containing protein 1; CCL7, C-C motif chemokine 7; CXCL9, C-X-C motif chemokine 9; HGF, Hepatocyte growth factor; IL6, Interleukin-6; NPX, Normalized Protein eXpresstion; TNFRSF9, Tumor necrosis factor receptor superfamily member 9; TNFRSF11B, Tumor necrosis factor receptor superfamily member 11B.

**
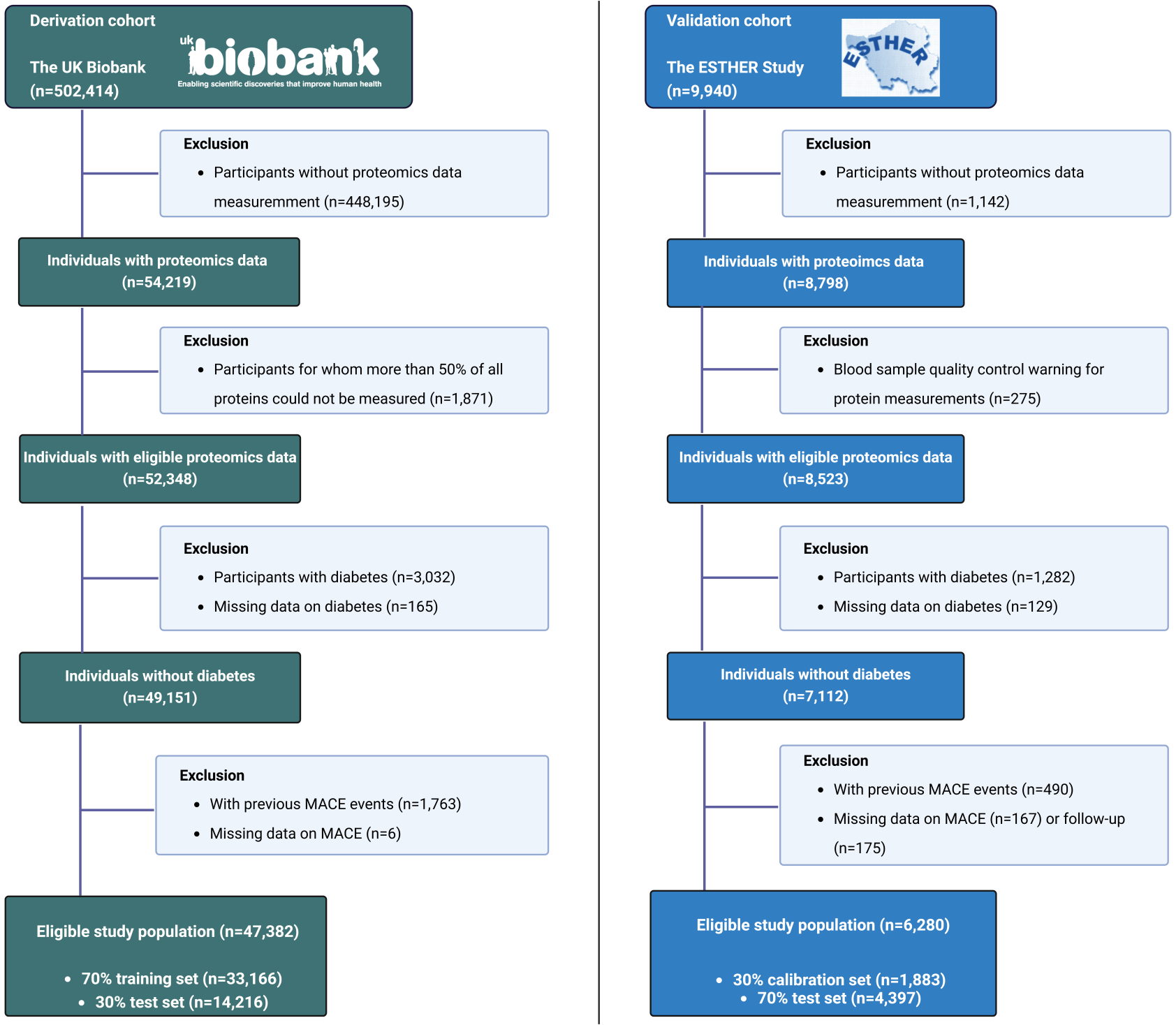
**

# **Supplemental** **Figure S1.** Flow-charts for participant inclusion and exclusion


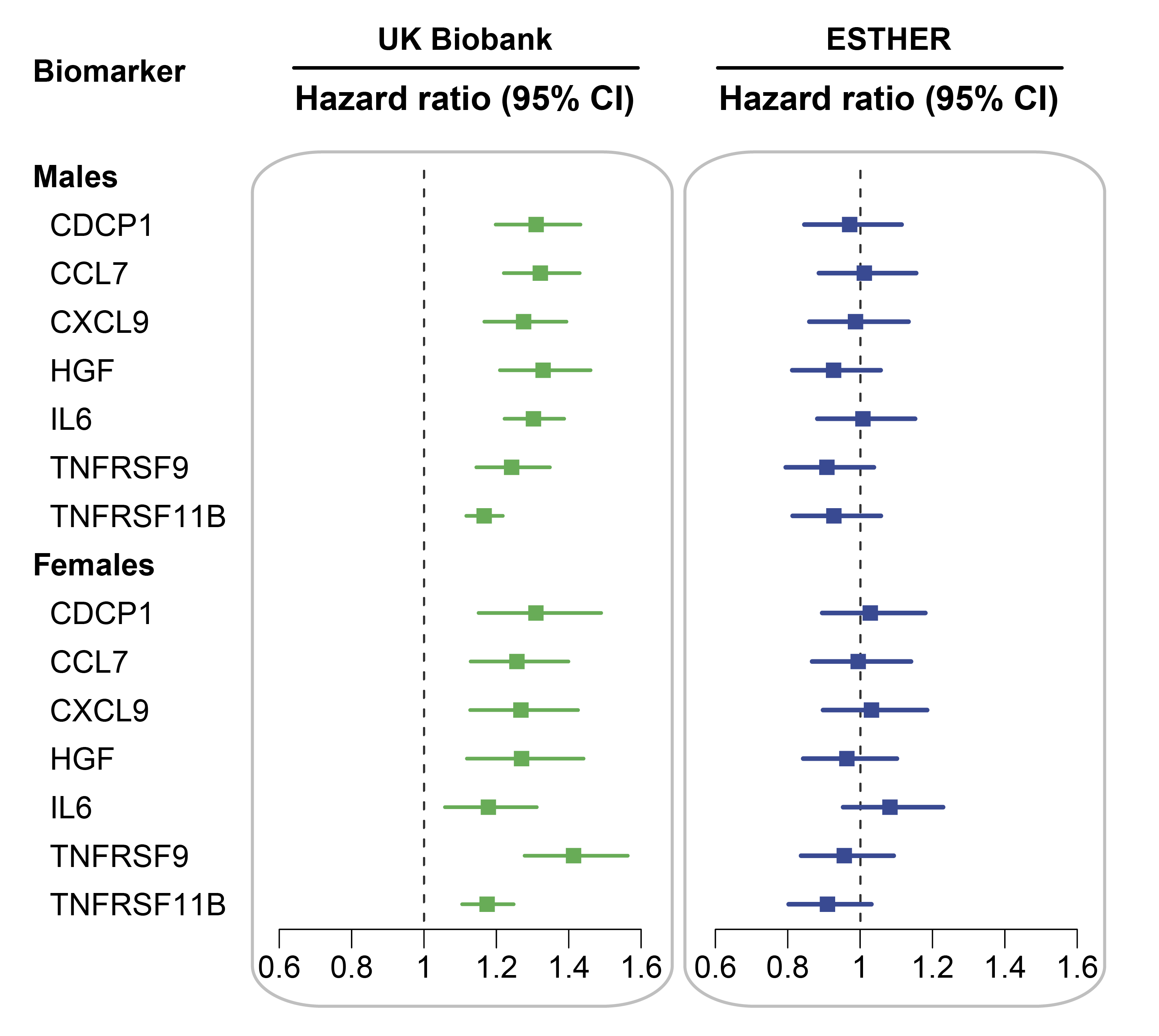


# **Supplemental Figure S2.** Associations between selected proteins and major cardiovascular events across sexes in the internal validation (30% of UK Biobank, N=14,216) and external validation (70% of ESTHER, N=4,397)

Note: The full names of the proteins are detailed in **Supplemental Table S1**.

**
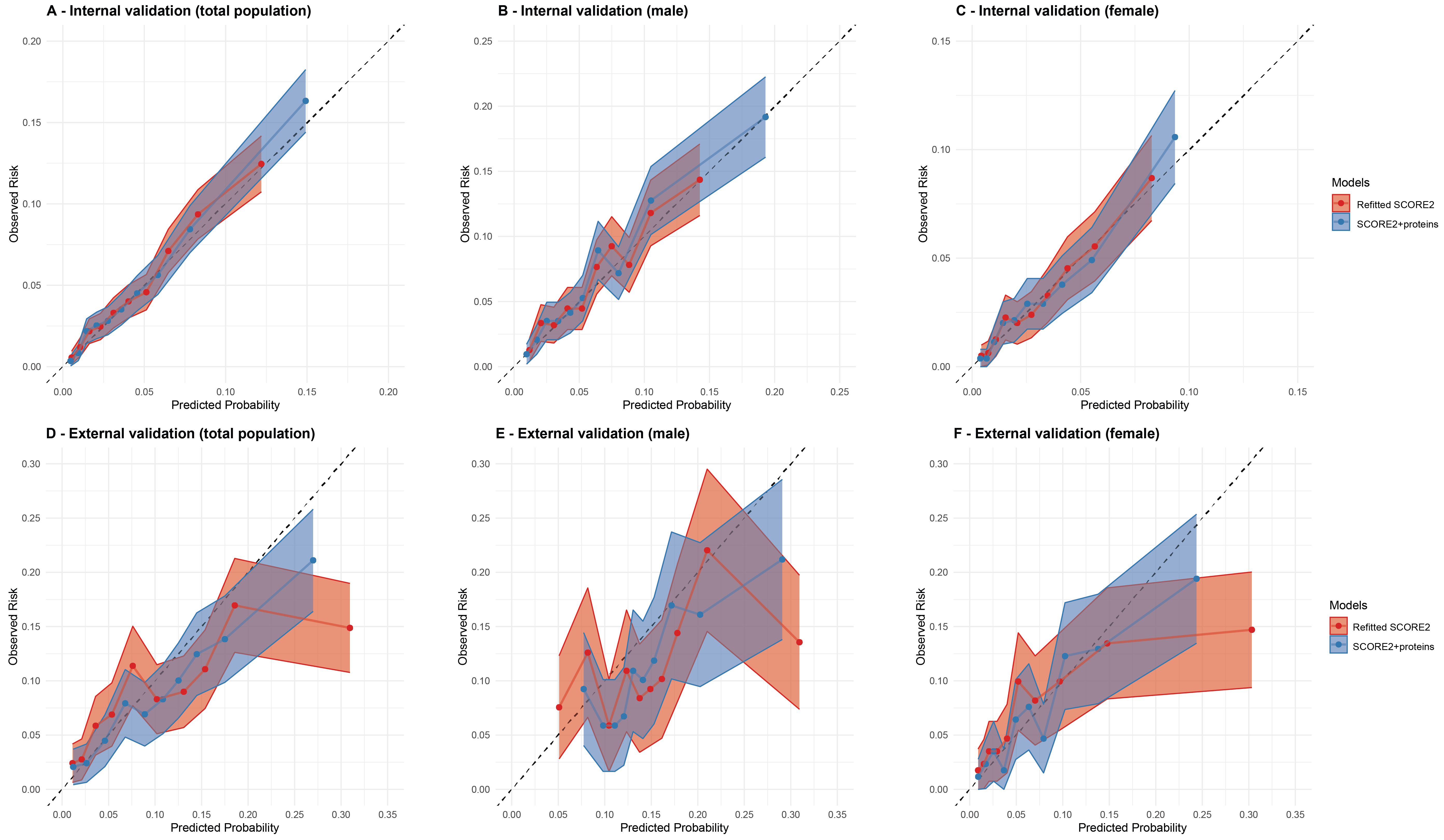
**

# **Supplemental Figure S3.** Calibration curves of the SCORE2 model with and without proteomics data for 10-year MACE risk prediction in the internal validation (30% of UK Biobank, N=14,216) and external validation (70% of ESTHER, N=4,397)

Proteins that were included in the SCORE2 model are detailed in **Supplemental Table S1**.

**
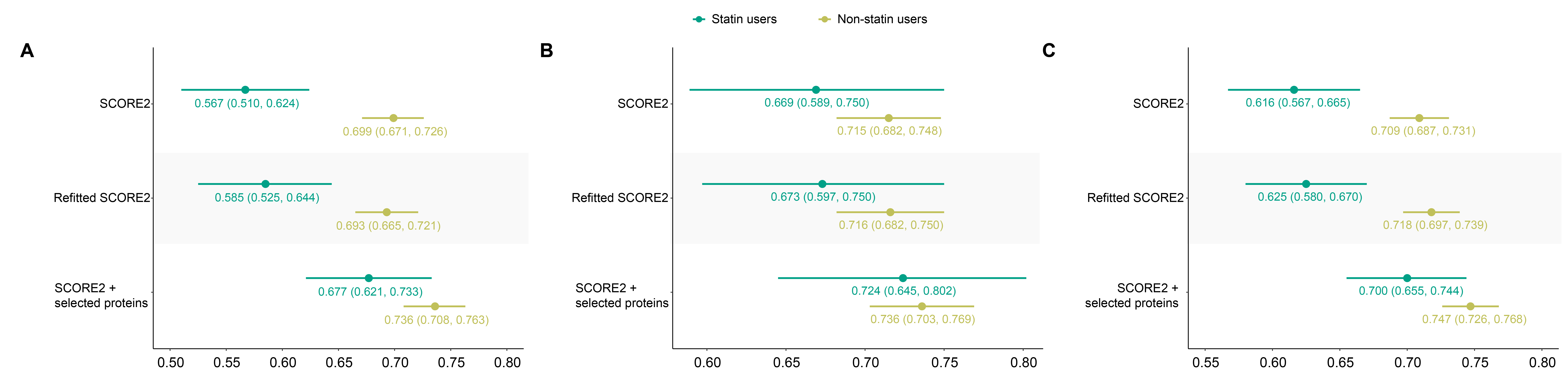
**

**Supplemental Figure S4.** Performance of the SCORE2 model and its extension with inflammation-related proteins stratified by statin use in the internal validation (30% of UK Biobank, N=14,216)

C-statistics (with 95% confidence intervals) for three models: original SCORE2, refitted SCORE2, and the SCORE2 model extended with selected inflammation-related proteins are shown stratified by statin use. The results are presented for males (**Panel A**), females (**Panel B**), and the total population (**Panel C**) in the UK Biobank validation set.

**References to Supplemental Materials**

1. Wik L, Nordberg N, Broberg J, Björkesten J, Assarsson E, Henriksson S, Grundberg I, Pettersson E, Westerberg C, Liljeroth E: **Proximity extension assay in combination with next-generation sequencing for high-throughput proteome-wide analysis**. *Mol Cell Proteomics* 2021, **20**.

2. Assarsson E, Lundberg M, Holmquist G, Björkesten J, Bucht Thorsen S, Ekman D, Eriksson A, Rennel Dickens E, Ohlsson S, Edfeldt G: **Homogenous 96-plex PEA immunoassay exhibiting high sensitivity, specificity, and excellent scalability**. *PLoS One* 2014, **9**(4):e95192.

3. Lundberg M, Eriksson A, Tran B, Assarsson E, Fredriksson S: **Homogeneous antibody-based proximity extension assays provide sensitive and specific detection of low-abundant proteins in human blood**. *Nucleic Acids Res* 2011, **39**(15):e102-e102.

4. Sun BB, Chiou J, Traylor M, Benner C, Hsu Y-H, Richardson TG, Surendran P, Mahajan A, Robins C, Vasquez-Grinnell SG: **Plasma proteomic associations with genetics and health in the UK Biobank**. *Nature* 2023, **622**(7982):329-338.

5. Dhindsa RS, Burren OS, Sun BB, Prins BP, Matelska D, Wheeler E, Mitchell J, Oerton E, Hristova VA, Smith KR: **Rare variant associations with plasma protein levels in the UK Biobank**. *Nature* 2023, **622**(7982):339-347.

6. Trares K, Bhardwaj M, Perna L, Stocker H, Petrera A, Hauck SM, Beyreuther K, Brenner H, Schöttker B: **Association of the inflammation-related proteome with dementia development at older age: results from a large, prospective, population-based cohort study**. *Alzheimers Res Ther* 2022, **14**(1):128.

7. Trares K, Wiesenfarth M, Stocker H, Perna L, Petrera A, Hauck SM, Beyreuther K, Brenner H, Schöttker B: **Addition of inflammation-related biomarkers to the CAIDE model for risk prediction of all-cause dementia, Alzheimer's disease and vascular dementia in a prospective study**. *Immun Ageing* 2024, **21**(1):23.

8. Niersmann C, Carstensen-Kirberg M, Maalmi H, Holleczek B, Roden M, Brenner H, Herder C, Schöttker B: **Higher circulating omentin is associated with increased risk of primary cardiovascular events in individuals with diabetes**. *Diabetologia* 2020, **63**(2):410-418.
